# Enhanced Cross-Reactive and Polyfunctional Effector-Memory T Cell Responses by ICVAX—a Human PD1-Based Bivalent HIV-1 Gag-p41 Mosaic DNA Vaccine

**DOI:** 10.1128/jvi.02161-21

**Published:** 2022-03-17

**Authors:** Samantha M. Y. Chen, Yik Chun Wong, Lok Yan Yim, Haoji Zhang, Hui Wang, Grace Chung Yan Lui, Xin Li, Xian Tang, Lin Cheng, Yanhua Du, Qiaoli Peng, Jinlin Wang, Hau-yee Kwok, Haode Huang, Thomas Tsz-Kan Lau, Denise Pui Chung Chan, Bonnie Chun Kwan Wong, Li Liu, Lisa A. Chakrabarti, Shui Shan Lee, Zhiwei Chen

**Affiliations:** a AIDS Institute, Department of Microbiology, State Key Laboratory of Emerging Infectious Diseases, Li Ka Shing Faculty of Medicine, The University of Hong Kong, Hong Kong SAR, People’s Republic of China; b Department of Veterinary Medicine, Foshan University, Foshan, People’s Republic of China; c HKU-AIDS Institute Shenzhen Research Laboratory and AIDS Clinical Research Laboratory, Guangdong Key Laboratory of Emerging Infectious Diseases, Shenzhen Key Laboratory of Infection and Immunity, Shenzhen Third People’s Hospital, Shenzhen, People’s Republic of China; d Department of Medicine and Therapeutics, The Chinese University of Hong Kong, Prince of Wales Hospital, Shatin, Hong Kong; e Stanley Ho Centre for Emerging Infectious Diseases, Postgraduate Education Centre, The Chinese University of Hong Kong, Prince of Wales Hospital, Shatin, Hong Kong; f Control of Chronic Viral Infection Group, Virus and Immunity Unit, Institut Pasteur, Paris, France; g Centre National de la Recherche Scientifique, UMR 3569, Paris, France; Emory University

**Keywords:** DNA vaccines, HIV-1, PD1, T cells

## Abstract

Vaccine-induced protective T cell immunity is necessary for HIV-1 functional cure. We previously reported that rhesus PD1-Gag-based DNA vaccination sustained simian-human immunodeficiency virus (SHIV) suppression by inducing effector-memory CD8^+^ T cells. Here, we investigated a human PD1-Gag-based DNA vaccine, namely, ICVAX, for clinical translation. PD1-based dendritic cell targeting and mosaic antigenic designs were combined to generate the ICVAX by fusing the human soluble PD1 domain with a bivalent HIV-1 Gag-p41 mosaic antigen. The mosaic antigen was cross-reactive with patients infected with B, CRF07/08_BC, and CRF01_AE variants. In mice, ICVAX elicited stronger, broader, and more polyfunctional T cell responses than mosaic Gag-p41 alone, and suppressed EcoHIV infection more efficiently. In macaques, ICVAX elicited polyfunctional effector-memory T cell responses that targeted multiple nonoverlapping epitopes of the Gag-p41 antigen. Furthermore, ICVAX manufactured following good manufacturing practices proved potent immunogenicity in macaques after biannual homologous vaccination, warranting clinical evaluation of ICVAX as an immunotherapy against HIV-1.

**IMPORTANCE** This study presents that ICVAX, a PD1-based DNA vaccine against HIV-1, could induce broad and polyfunctional T cell responses against different HIV-1 subtypes. ICVAX encodes a recombinant antigen consisting of the human soluble PD1 domain fused with two mosaic Gag-p41 antigens. The mosaic antigens cover more than 500 HIV-1 strains circulating in China including the subtypes B/B’, CRF01_AE, and CRF07/08_BC. In mice, ICVAX elicited stronger, broader, and more polyfunctional T cell responses, with better EcoHIV suppression than the nontargeting mosaic Gag-p41 DNA vaccine. Moreover, both lab-generated and GMP-grade ICVAX also elicited strong polyfunctional effector-memory T cell responses in rhesus macaques with good immunogenicity against multiple nonoverlapping epitopes of the Gag-p41 antigen. This study therefore highlights the great potential to translate the PD1-based DNA vaccine approach into clinical use, and opens up new avenues for alternative HIV-1 vaccine design for HIV-1 preventive and functional cure.

## INTRODUCTION

The vast genetic diversity of human immunodeficiency virus 1 (HIV-1) represents a major challenge for the development of prophylactic and therapeutic vaccines against this highly prevalent virus. Currently, over 38 million people are living with HIV-1 infection, with about 67% of the patients receiving combination antiretroviral therapy (cART) (1). The cART potently suppresses viral load and prevents the onset of AIDS in infected patients. However, lifelong cART treatment is required to prevent viral rebound, and this creates a huge financial burden particularly in developing countries. The toxicity of cART drugs could also be a concern in long-term treatment (2). Immunotherapies aiming at long-term suppression of viral replication without the use of cART are being actively explored in the AIDS research field to develop a functional HIV-1 cure (2).

CD8^+^ T cells are directly involved in suppressing HIV-1 viremia in patients and simian immunodeficiency virus (SIV) infection in the macaque model of AIDS (3, 4). Polyfunctional T cell responses are more commonly found in HIV-1 patients with better viral control, especially in elite controllers, than in progressor patients (5, 6). Based on these findings, multiple therapeutic vaccination strategies have been tested for their ability to induce protective T cell responses in animal models and in patients, with the aim of achieving a functional HIV-1 cure. Vaccination strategies involve the use of novel vaccine vectors, including rhesus cytomegalovirus (CMV) vectors, and multiple vector combinations for the induction of potent anti-HIV-1 T cell responses (7, 8). Another strategy utilizes unique recombinant antigen designs, including conserved antigens (9) and recombinant mosaic antigens designed *in silico* to target multiple viral subtypes (8, 10, 11), as well as engineered antigens targeted by elite controllers who control HIV-1 infection without cART (12). A third strategy involves the use of latency reversing agents (LRA) combined to vaccination for the elimination of latent viral reservoirs in a “shock and kill” approach (13, 14).

We have previously explored two individual strategies to enhance the immunogenicity and efficacy of HIV-1 T cell vaccines. The first strategy focuses on the modification of antigens encoded by a DNA vaccine, in which the target antigen is genetically fused to a soluble program-death 1 (PD1) domain (15). This construct allows an efficient delivery of the fusion antigen to dendritic cells (DC) via interactions between PD1 and its ligands (PD-L1 and PD-L2) constitutively expressed on DC. The fused antigens could thus be captured for cross-presentation by DC to prime potent CD8^+^ T cell responses (15, 16). We recently demonstrated broad T cell immunogenicity of a DNA vaccine encoding a macaque PD1 domain fused to the p27 Gag capsid antigen of SIV (rhPD1-p27) in rhesus macaques. Furthermore, the rhPD1-p27 vaccine induced complete viral control in challenged animals, with undetectable viremia in vaccinated macaques reached 2–3 months after a high-dose intravenous challenge with a pathogenic simian-human immunodeficiency virus (SHIV) (17). These studies demonstrated the potential of the PD1-based DNA vaccination for a functional HIV-1 cure, which led us to develop a candidate human PD1-Gag-based DNA vaccine that would be applicable to patients.

We also explored how to enhance the breadth and coverage of target antigens using mosaic antigenic constructs. This approach maximizes the coverage of potential T cell epitopes (PTE) across diverse viral variants (18). We have designed and constructed two mosaic antigens targeting the HIV-1 Gag-p41 antigen, comprising the p17 matrix and the p24 capsid. The two Gag-p41 constructs were expressed either separately from conventional DNA plasmids or together as a genetically linked bivalent antigen expressed in a modified vaccinia virus TianTan (MVTT) vector in rodent models (19). HIV-1 Gag-p41 was chosen as the target because the magnitude and the breadth of functional T cell response against Gag, but not other HIV-1 proteins, are associated with better HIV-1 control (20–22). Our mosaic constructs were designed *in silico* (23), based on >500 HIV-1 viral strains circulating in China, and covered HIV-1 subtypes B/B’, CRF01_AE, and CRF07/08_BC. The two mosaic antigens together showed a >95% match for at least 7 of 9 amino acids in all the PTE tested (19). The two mosaic gp41 constructs were sufficiently immunogenic to induce T cell responses against all the HIV-1 subtypes tested in mice, when expressed from two conventional DNA plasmids, or in combination from an MVTT vector (19).

In the present study, PD1-based dendritic cell targeting and mosaic antigenic designs were combined to generate a PD1-based DNA vaccine called ICVAX. The ICVAX vaccine encodes a recombinant antigen consisting of the human soluble PD1 domain fused to the two mosaic Gag-p41 antigens. ICVAX immunogenicity and its efficacy in controlling an EcoHIV challenge were examined in mice. The ICVAX vaccine, produced in the laboratory and according to good manufacturing practices (GMP), was then further evaluated for immunogenicity in rhesus macaques.

## RESULTS

### T cell reactivity against the two HIV-1 mosaic Gag-p41 antigens in HIV-1 patients.

The two HIV-1 mosaic Gag-p41 antigens, namely, mos1 and mos2 (19), were designed to cover the diversity of viral strains circulating in China and many other countries. The mosaic antigen design algorithm generated recombinant antigens with optimized PTE coverage against the input sequences (18). However, it remained unclear whether these engineered antigens were sufficiently immunogenic to reactivate T cells from patients. Since the goal of our ICVAX vaccine was to stimulate therapeutic T cell immunity, we evaluated the T cell reactivity against the two mosaic antigens in the cART-treated or cART-naive patient cohort who are mostly Chinese male (Table 1).

**TABLE 1:** Clinical characteristics of cART-naive and cART-treated patients^*a*^

Characteristic	cART-naïve (*n *= 44)	cART-treated (*n *= 39)	*P* value
Demographic			
Age, median yrs (interquartile range)	35 (29–38)	45 (35–55)	0.0001^*b*^
Gender (Male)	37 (84.1%)	35 (89.7%)	0.526
Ethnicity (Chinese, Non-Chinese Asian)	41 (93.2%), 3 (6.81%)	37 (94.9%), 2 (5.13%)	1.000
Transmission route			
Heterosexual	14 (31.8%)	19 (48.7%)	0.177
Homosexual	25 (56.8%)	18 (46.1%)	0.383
Bisexual	5 (11.4%)	1 (2.56%)	0.207
Injection drug use	0 (0%)	1 (2.56%)	0.470
Correlation between age and IFN-γ response in total HIV-1 patients			
Total patient age vs mos1	r = −0.0770		0.489
Total patient age vs mos2	r = 0.0235		0.833
Blood test			
Mean CD4 count (cells/mm^3^ ± std. error mean)	422.3 ± 22.35	638.3 ± 55.18	<0.0001^*b*^
Mean viral load (copies/mL ± std. error mean)	846129 ± 195859	17.21 ± 16.66	<0.0001^*b*^
Concurrent infection			
HBV	2 (4.55%)	1 (2.56%)	1.000
HCV	1 (2.27%)	0 (0%)	1.000
Syphilis	9 (20.5%)	5 (12.8%)	0.390
Antiretroviral regimen received			
Two NRTI + one INSTI	N/A	11 (27.5%)	N/A
Two NRTI + one NNRTI	N/A	22 (55.0%)	N/A
Two NRTI + one PI / one PI with booster	N/A	6 (15.0%)	N/A

a Clinical characteristics of patients with PBMC evaluated for IFN-g response against the mos1 and mos2 antigens were summarized. Each parameter was statistically compared between the two patient groups with *P* < 0.05 considered as statistically significant. NRTI, nucleoside reverse transcriptase inhibitor; INSTI, integrase strand transfer inhibitor; NNRT, non-nucleoside reverse transcriptase inhibitor; PI, protease inhibitor.

b *P *≤ 0.05 was considered as statistically significant.

**FIG 1:**
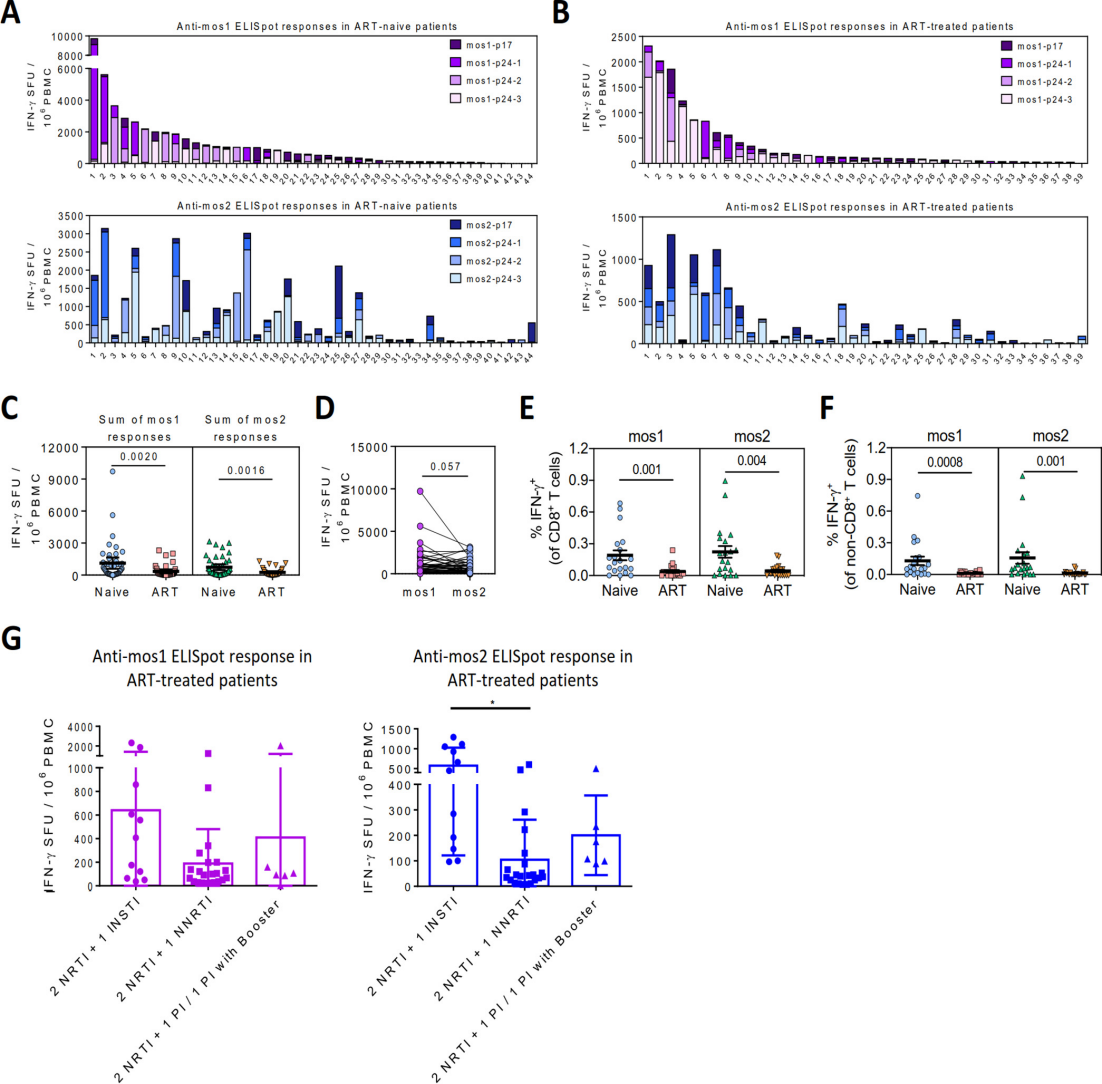
T cell reactivity of the bivalent mosaic HIV-1 Gag-p41 antigen in cART-naive or cART-treated HIV-1 patients. (A–B) T cell reactivity against peptide pools covering the mosaic HIV-1 Gag-p41 antigen 1 (mos1; top) and mosaic antigen 2 (mos2; bottom) from individual cART-naive (A) or cART-treated patients (B), as measured by IFN-γ ELISpot assays. Each bar represents total IFN-γ responses of an individual patient. SFU, spot-forming units. (C) Comparisons of the total ELISpot responses against mos1 and mos2 between cART-naive and cART-treated patients. (D) Comparisons of the total ELISpot responses between the mos1 and mos2 antigens in all study patients. (E–F) CD8^+^ (E) and non-CD8^+^ (F) T cell responses against the mos1 and mos2 antigen between cART-naive and cART-treated patients, as measured by intracellular cytokine staining (ICS) assays. (G) Patients were categorized based on the combination and the class of cART drugs they received. Response of IFN-γ of each group were then statistically compared.

T cell antigenicity was first evaluated by interferon-γ (IFN-γ) ELISpot assays. Two sets of 15mer peptides were synthesized, with 11 overlapping amino acids spanning the mos1 or mos2 antigens separately. For each peptide set, individual peptides were subdivided into 4 nonoverlapping peptide pools, with one spanning Gag-p17 (P17) and three spanning Gag-p24 (P24-1, P24-2, and P24-3). These peptide pools were used to stimulate *ex vivo* peripheral blood mononuclear cells (PBMC) isolated from either cART-naive (*n *= 44) or cART-treated (*n *= 39) HIV-1 patients (Fig. 1A to D and Table 1). Overall, most HIV-1-infected patients demonstrated T cell reactivity against at least one of the two mosaic antigens. All eight individual peptide pools from either mosaic antigens were sufficiently immunogenic to elicit T cell responses in cART-naive (mos1: 41/44, 93%; mos2: 43/44, 98%) or cART-treated (mos1: 38/39, 97%; mos2: 39/39, 100%) patients, thus illustrating the broad antigenicity of the mosaic antigens. The genetic diversity of both the patients and the virus in the patients could account for the unique pattern of T cell reactivity toward individual mosaic peptide pools in each patient (Fig. 1A and B). The results also indicated that T cell responses within a diverse patient population predominantly targeted p24 Gag over p17 Gag, but did not target any particular p24 regions of the mos1 and mos2 antigens (Fig. 1A and B).

Within the cART-naive patient cohort, the median IFN-γ^+^ spot forming units (SFU) against mos1 and mos2 were 562 SFU/10^6^ PBMC (range: 0–9717 SFU/10^6^ PBMC) and 315 SFU/10^6^ PBMC (range: 0–3145 SFU/10^6^ PBMC), respectively (Fig. 1C). In comparison, T cell responses against mos1 and mos2 in cART-treated patients had a median of 113 SFU/10^6^ PBMC (range: 0–2313 SFU/10^6^ PBMC) and 99 SFU/10^6^ PBMC (range: 7–1290 SFU/10^6^ PBMC), respectively (Fig. 1C). Moreover, significantly higher T cell reactivities against mos1 and mos2 were detected from cART-naive patients (Fig. 1C). Since CD8^+^ T cell response is better in younger individuals (24, 25), we evaluated whether the two patient groups had any age differences. Although cART-naive patients were significantly younger, there was no correlation between age and IFN-γ responses against mos1 or mos2 in total HIV-1 patients, supporting that higher T cell responses observed in cART-naive patients were unlikely due to age differences (Table 1). Moreover, there was a trend of stronger reactivity of the mos1 antigen over the mos2 antigen, although not statistically significant (Fig. 1D). Overall, the results suggested a maintenance of antiviral T cell effector responses by ongoing viral replication.

Further analysis was performed to delineate the CD8^+^ and CD4^+^ T cell reactivity against the two mosaic antigens. Patients’ PBMC from cART-naive and cART-treated HIV-1 patients were stimulated with complete mos1 and mos2 overlapping peptide pools *ex vivo*, and IFN-γ expression from CD8^+^ and non-CD8^+^ T cells (mainly CD4^+^) were determined by intracellular cytokine staining (ICS). After mos1 and mos2 peptide pool stimulation, IFN-γ^+^ cells were detected in either CD8^+^ T cell or both CD8^+^ and non-CD8^+^ T cell populations in most patients (Fig. 1E and F). Significantly higher T cell responses specific to mos1 and mos2 were detected in cART-naive patients compared to those that received cART treatment in both CD8^+^ and non-CD8^+^ populations (Fig. 1E and F).

**FIG 2:**
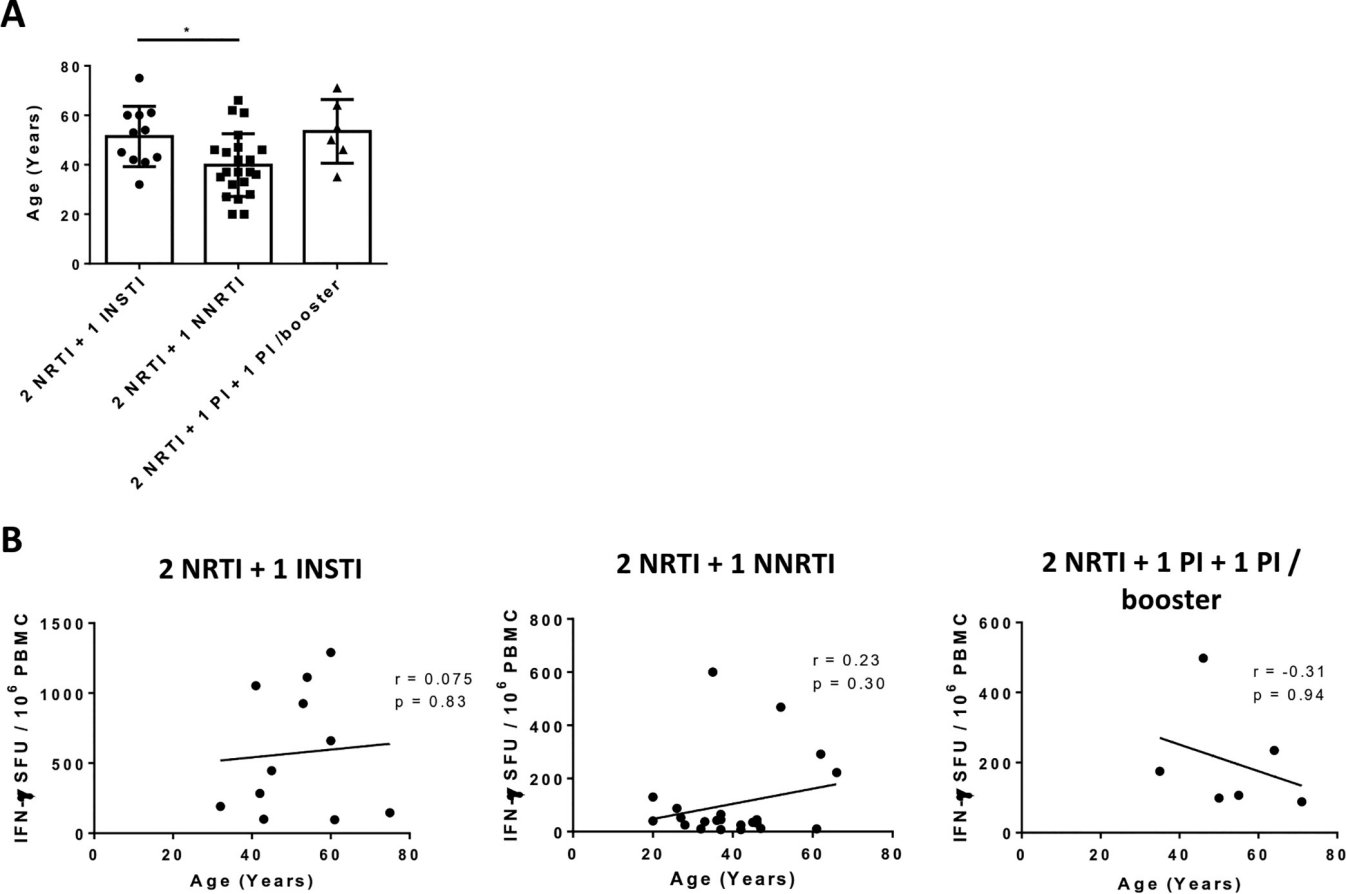
Age differences of patients with different treatment regiments and their correlation with IFN-γ responses against mos2. (A) The ages of patients receiving different treatment regiments were compared and (B) correlated with IFN-γ response against mos2 evaluated by IFN-γ ELISpot assays. NRTI, nucleoside reverse transcriptase inhibitor; NNRTI, non-nucleoside reverse transcriptase inhibitor; INSTI, integrase strand transfer inhibitors; PI, protease inhibitor.

Patients were also categorized according to the class of cART drugs they received. IFN-γ responses against mos1 and mos2 between different treatment regimens (Table 1) were statistically compared. Results showed that patients receiving a combination treatment of 2 nucleoside reverse transcriptase inhibitor and 1 integrase strand transfer inhibitor (2 NRTI + 1 INSTI) had a higher IFN-γ response against mos2 than patients receiving 2 nucleoside reverse transcriptase inhibitor plus 1 non-nucleoside reverse transcriptase inhibitor (2 NRTI + 1 NNRTI) (Fig. 1G). However, plasma viral load of all patients under the 2 NRTI + 1 NNRTI treatment regimen was below the detection limit (Table 2), indicating that patients on NNRTI had achieved good antiviral control. Although there was an age difference between the 2 NRTI + 1 INSTI and 2 NRTI + 1 NNRTI treatment groups, there was no correlation between age and IFN-γ response against mos2 in these patients (Fig. 2). Collectively, our results demonstrated that both mos1 and mos2 antigens were antigenic in HIV-1 patients, and these mosaic antigens contain epitopes that are readily recognized by T cells from our local HIV-1 patients infected with B, CRF07/08_BC, or CRF01_AE variants (26, 27). These data support the use of these mosaic antigens for further vaccine development.

**TABLE 2:** Plasma viral load of patients in different cART treatment groups^*a*^

Antiviral regimen received^*a*^	Patient code	Viral load (copies/mL)
Two NRTI + one INSTI	1	Below detection limit
	3	Below detection limit
	5	650
	7	Below detection limit
	8	21
	9	Below detection limit
	14	Below detection limit
	20	Below detection limit
	29	Below detection limit
	31	Below detection limit
	32	Below detection limit
Two NRTI + one NNRTI	4	Below detection limit
	6	Below detection limit
	10	Below detection limit
	11	Below detection limit
	12	Below detection limit
	13	Below detection limit
	17	Below detection limit
	18	Below detection limit
	19	Below detection limit
	22	Below detection limit
	23	Below detection limit
	24	Below detection limit
	27	Below detection limit
	28	Below detection limit
	30	Below detection limit
	33	Below detection limit
	34	Below detection limit
	35	Below detection limit
	36	Below detection limit
	37	Below detection limit
	38	Below detection limit
	39	Below detection limit
Two NRTI + one PI / one PI with booster	2	Below detection limit
	16	Below detection limit
	21	Below detection limit
	25	Below detection limit
	26	Below detection limit
	40	Below detection limit

a Patients were categorized according to the drug class of their cART treatment, and their plasma viral load is indicated. NRTI: nucleoside reverse transcriptase inhibitor; INSTI: integrase strand transfer inhibitor; NNRTI: non-nucleoside reverse transcriptase inhibitor; PI: protease inhibitor.

### Design of ICVAX, a PD1-based HIV-1 mosaic DNA vaccine.

**FIG 3:**
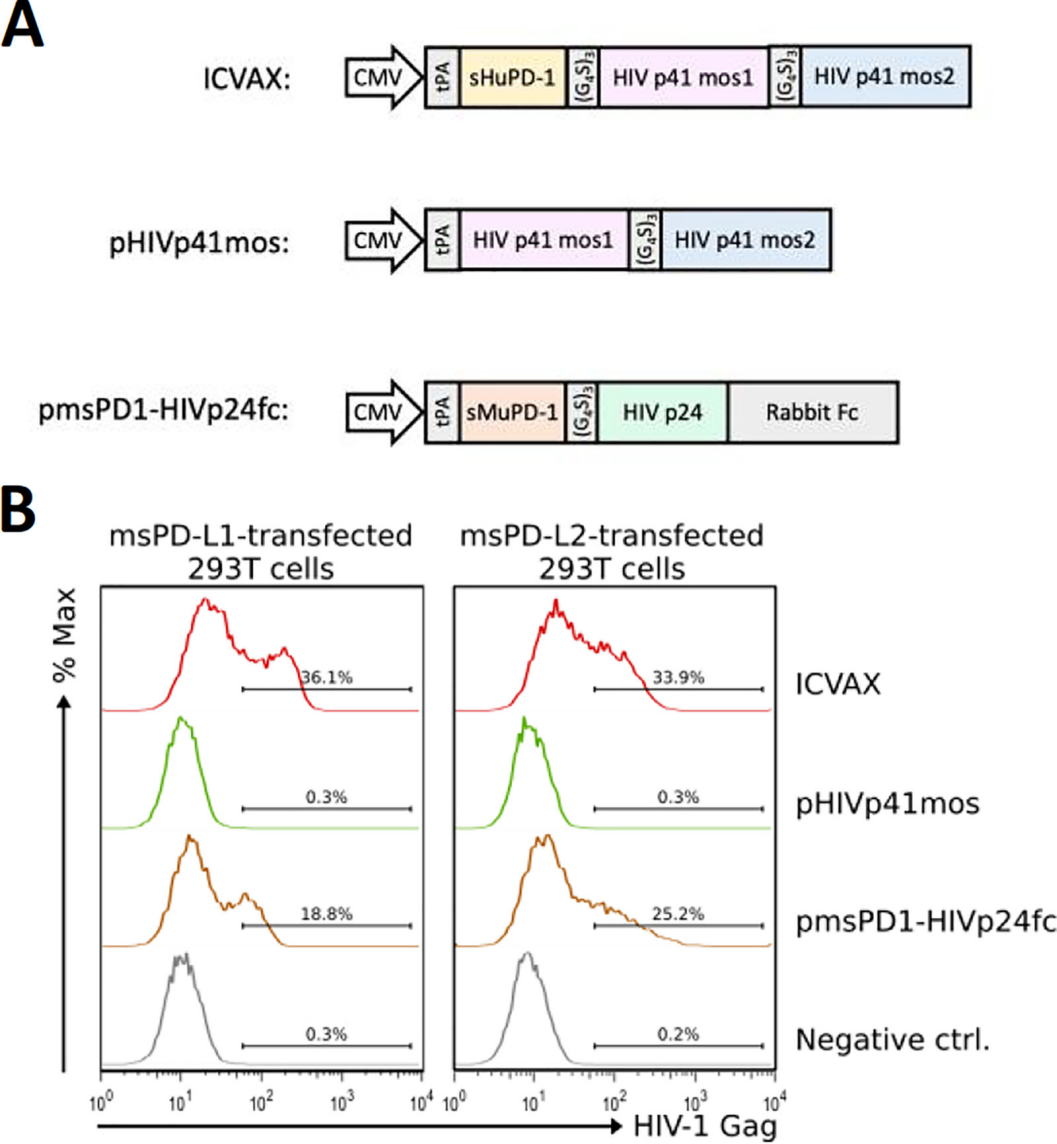
Validation of PD1-based DNA vaccine constructs encoding the bivalent HIV-1 mos1 and mos2 antigens. (A) Designs of the PD1-based ICVAX DNA vaccine and other DNA vaccines tested in this study. (B) Flow cytometry histograms showing the binding of the antigens encoded by the DNA vaccines to 293T cells that transiently expressed the PD1 ligands PD-L1 and PD-L2.

To generate a PD1-based HIV-1 mosaic DNA vaccine, we designed and generated a codon-optimized antigen expression construct consisting of a human soluble PD1 domain fused to mos1 and mos2 antigens (Fig. 3A). A DNA sequence encoding the tissue plasminogen activator (tPA) leader sequence was added at the 5′-end of the expression construct to direct the expressed fusion antigen for secretion. Moreover, DNA sequences encoding a flexible linker (G_4_S)_3_ sequence were added between the soluble PD1 domain and mos1 sequences, as well as between mos1 and mos2 sequences to assist proper protein folding. This antigen expression construct was cloned into a pVAX1 plasmid vector to generate the ICVAX vaccine, placing the construct under the control of a ubiquitously expressed CMV promoter (15). A non-PD1 control DNA vaccine, namely, pHIVp41mos, which only encoded the bivalent mosaic antigen, was also generated for the purpose of comparison.

We first determined the ability of the recombinant antigens expressed from these DNA vaccines to bind to PD1 ligands. To this goal, 293T cells were transiently transfected with ICVAX or pHIVp41mos. A previously tested PD1-based DNA vaccine, pmsPD1-HIVp24fc, which encoded a murine soluble PD1-fused to the HIV-1 p24 antigen (15), was also included as a positive control. Supernatants obtained 2 days after transfection were incubated with 293T cells that transiently expressed murine PD-L1 or PD-L2, respectively. These ligands are known to cross-react with human PD1. The binding of the recombinant antigens to the PD1 ligand-expressing cells was detected by cell surface staining with anti-Gag antibody followed by flow cytometry analysis. The findings showed that the human PD1-fused bivalent mosaic antigen expressed from the ICVAX bounded to murine PD-L1 and PD-L2 expressed on the transfected 293T cells with comparable levels to the murine PD1-fused p24 antigen expressed from pmsPD1-HIVp24fc (Fig. 3B). In contrast, the non-PD1 bivalent mosaic antigen from pHIVp41mos failed to show any binding to the PD-L1/L2 expressing cells, demonstrating the necessity of the soluble PD1 domain for targeting antigens toward PD1-ligand-expressing cells (e.g., dendritic cells). The data therefore illustrated the possibility of *in vivo* ICVAX testing in mice.

### Induction of cross-reactive T cell responses by ICVAX in mice.

**FIG 4:**
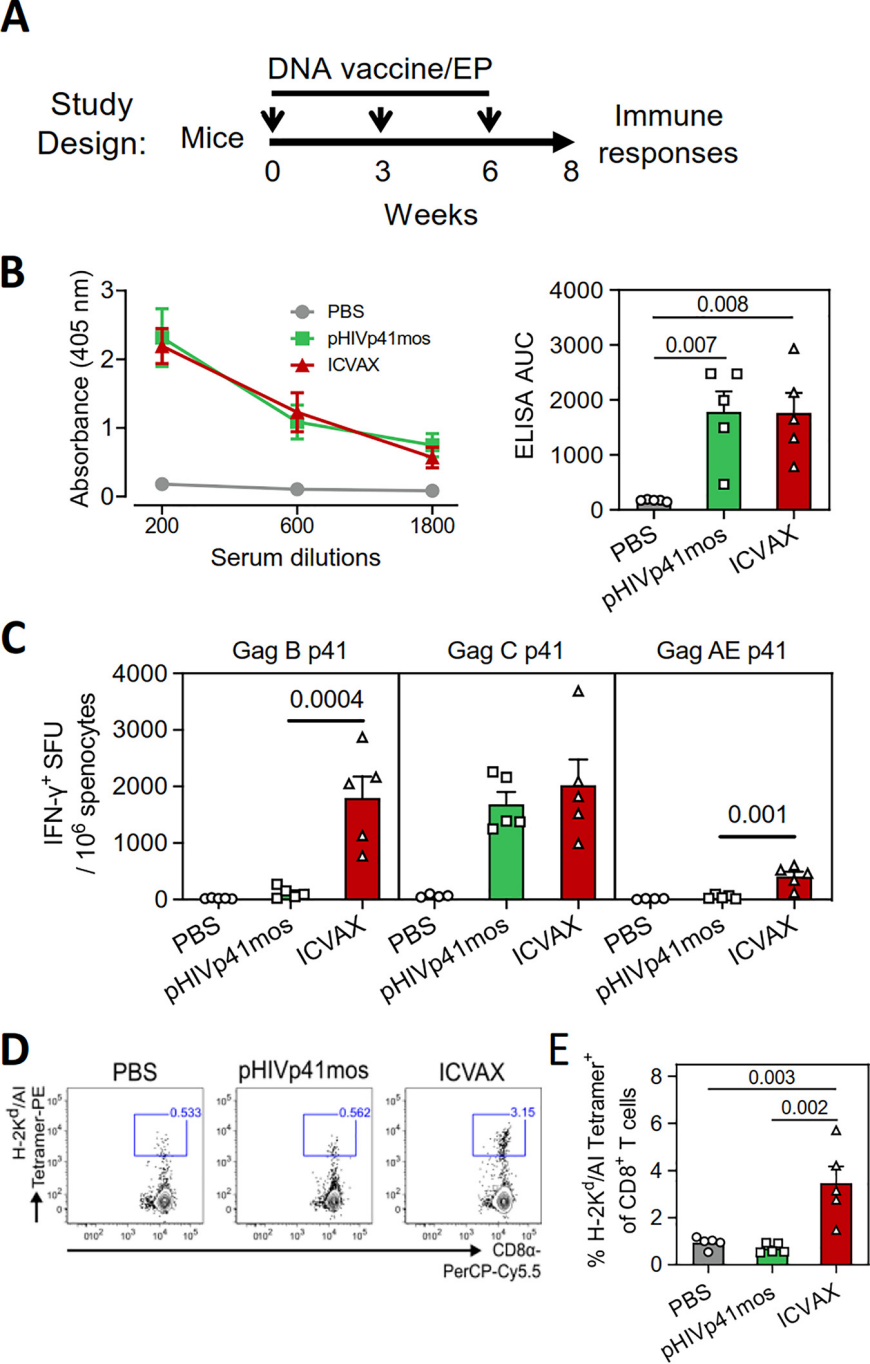
Immunogenicity of ICVAX vaccine in mice. (A) Schematic showing the immunization schedule of the DNA vaccines tested. In brief, BALB/c mice were immunized with 100 μg of the PD1 based ICVAX vaccine, the conventional pHIVp41mos vaccine, or with PBS three times in 3-week intervals via intramuscular (i.m.) injection with electroporation (EP). Antibody and T cell responses were measured at 2 weeks after the last immunization. (B) IgG antibody responses against HIV-1 Gag p24 were measured by ELISA. Anti-p24 antibodies in serial dilutions of sera were measured in absorbance (left panel) while anti-p24 titers were calculated as area under the curve (AUC) (right panel). (C) T cell responses against Gag-p41 peptide pools from HIV-1 subtypes B, C, and AE induced in the three groups, as measured by IFN-γ ELISpot assays. SFU, spot-forming units. (D) Representative flow cytometry plots showing the staining of splenic CD8^+^ T cells with the H-2K^d^/AI tetramer. (E) Comparisons of the frequencies of AI-specific CD8^+^ T cells induced among the three test groups, as measured by tetramer staining.

The immunogenicity of the ICVAX vaccine was evaluated *in vivo*. BALB/c mice were immunized with 100 μg of ICVAX, pHIVp41mos, or PBS alone by intramuscular injection with electroporation (i.m./EP) for three times at 3-week intervals (Fig. 4A). Comparable levels of anti-p24 IgG antibody titers were measured in serum at 2 weeks post the second immunization between the ICVAX and pHIVp41mos groups (Fig. 4B), demonstrating a similar capacity for antibody responses by these two vaccines.

**FIG 5:**
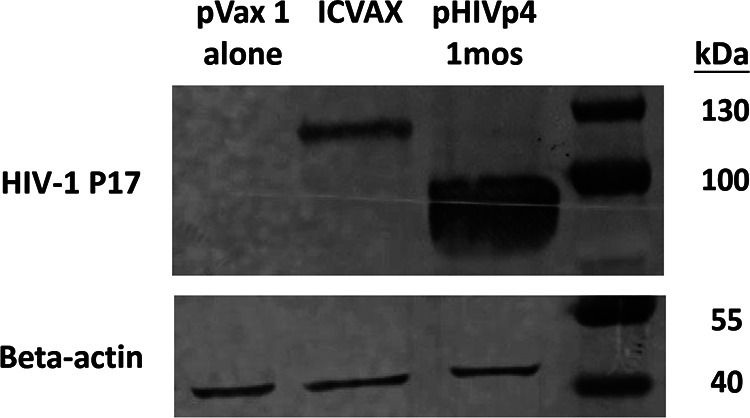
Expression of secretory ICVAX and pHIVp41mos. The expression of secretory ICVAX and pHIVp41mos was evaluated by transfecting 293T cells with the indicated DNA constructs. Supernatants were harvested at 48 h posttransfection, and the expression was detected by the anti-HIV-1 Gag-p17 antibody using Western blot. Size of ICVAX that harbors the soluble PD1 was 120 kDa, and pHIVp41mos was 82 kDa, respectively.

Splenic T cell responses were then measured at 2 weeks after the last immunization by sacrificing the animals to harvest enough splenocytes for functional assays, a time point where high magnitudes of T cell responses were induced by PD1-based DNA vaccines (15). T cell cross-reactivity against diverse Gag-p41 antigens derived from HIV-1 subtypes B/B’, CRF07/08_BC, and CRF01_AE was first determined by IFN-γ ELISpot assays. Importantly, ICVAX induced higher magnitudes of T cell responses against Gag-p41 from HIV-1 subtypes B and AE than pHIVp41mos (Fig. 4C). In contrast, T cell responses against HIV-1 subtype C Gag-p41 were similarly strong between these two vaccination groups. The more superior T cell response of ICVAX was not due to a better expression of the antigen from ICVAX (Fig. 5). These results indicated that ICVAX, but not pHIVp41mos, induced cross-reactive Gag-specific T cells against multiple viral subtypes.

Next, the H-2K^d^/AI tetramer containing the immunodominant AMQMLKDTI (AI) Gag epitope was used to directly detect antigen-specific CD8^+^ T cells from vaccinated mice. A significant increase of the frequency of tetramer^+^ cells within the splenic CD8^+^ T cell population was detected in the ICVAX group compared to the pHIVp41mos group (Fig. 4D and E). This confirmed that the antigen-specific CD8^+^ T cell response could be enhanced when the target antigen expressed from a DNA vaccine was genetically fused to a soluble PD1 domain as we previously reported (15, 16).

### Polyfunctionality analysis of T cell responses induced by ICVAX in mice.

**FIG 6:**
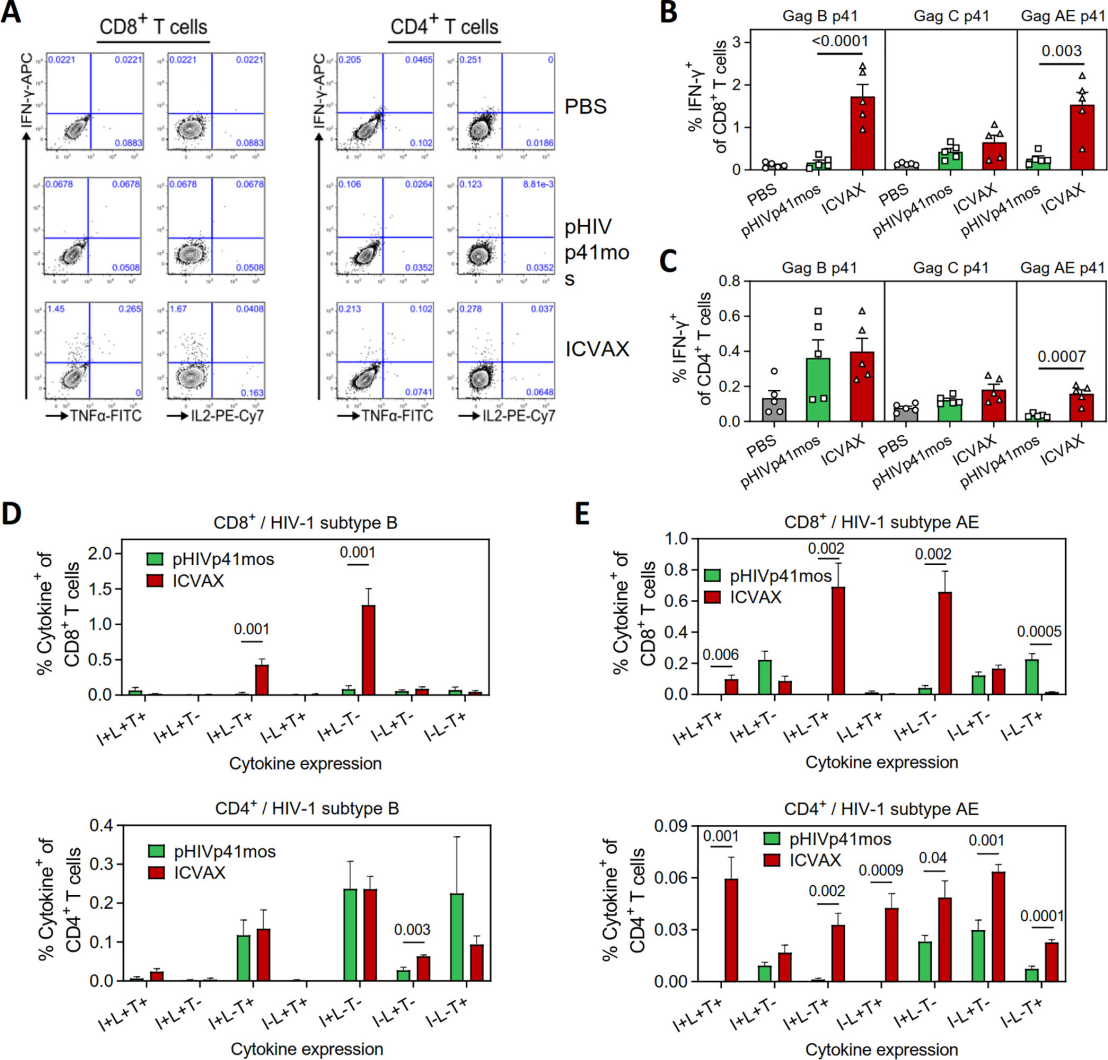
Analysis of T cell polyfunctionality induced by ICVAX vaccination in mice. (A) Representative flow cytometry plots showing the expression of IFN-γ, TNF-α, and IL-2 from splenic CD8^+^ (left panels) or CD4^+^ T cells (right panels) in mice immunized with PBS, pHIVp41mos, or ICVAX, after *ex vivo* stimulation with an HIV-1 subtype B Gag-p41 peptide pool. (B) Frequencies of IFN-γ-expressing CD8^+^ T cells against Gag-p41 peptide pools from HIV-1 subtypes B, C, or AE. (C) Frequencies of IFN-γ-expressing CD4^+^ T cells against Gag-p41 peptide pools from HIV-1 subtypes B, C, or AE. (D) Polyfunctionality analysis of CD8^+^ (top panel) and CD4^+^ T cells (bottom panel) against the HIV-1 subtype B Gag-p41 peptide pool. (E) Polyfunctionality analysis of CD8^+^ (top panel) and CD4^+^ T cells (bottom panel) against the HIV-1 subtype AE Gag-p41 peptide pool. For D and E: I, IFN-γ; L, IL-2; T, TNF-α.

The CD8^+^ and CD4^+^ T cell responses induced by ICVAX in mice were further examined by ICS to detect the expression of IFN-γ, tumor necrosis factor α (TNF-α) and interleukin 2 (IL-2). Consistent with the IFN-γ ELISpot results, ICVAX elicited higher frequencies of IFN-γ^+^ CD8^+^ T cells responding against Gag-p41 peptide pools from HIV-1 subtypes B and CRF01_AE than pHIVp41mos, while the responses against the subtype C Gag-p41 peptide pool was of comparable magnitude for these two vaccines (Fig. 6A and B). IFN-γ^+^ CD4^+^ T cell responses induced by ICVAX were significantly higher against Gag-p41 from subtype CRF01_AE compared to pHIVp41mos, while the responses induced against the other two HIV-1 subtypes were comparable (Fig. 6C).

Because the presence of polyfunctional Gag-specific T cells is associated with better HIV-1 control in patients (5, 6), we determined the polyfunctionality of T cell responses induced by ICVAX in mice from the same experiment, focusing on responses against HIV-1 subtypes B and AE that were significantly different between the two vaccine groups. Within the CD8^+^ T cell compartment, high frequencies of IFN-γ^+^TNF-α^+^ and IFN-γ^+^ cells were found exclusively in the ICVAX group after stimulation with Gag p41 for viral subtype B (Fig. 6D). In addition to these two functional T cell subsets, ICVAX also induced triple-positive IFN-γ^+^TNF-α^+^IL-2^+^ CD8^+^ T cell subset specifically against subtype AE Gag-p41 (Fig. 6E). Similarly, compared to the pHIVp41mos-vaccinated mice, ICVAX-vaccinated mice had higher frequencies of polyfunctional CD4^+^ T cells, including cells that coexpressed IFN-γ/TNF-α/IL-2, IFN-γ/TNF-α, and TNF-α/IL-2, specifically against subtype CRF01_AE Gag-p41 (Fig. 6E). These results demonstrated a higher T cell polyfunctionality was induced by the ICVAX vaccine.

### Efficacy of the ICVAX vaccination against an EcoHIV challenge in mice.

**FIG 7:**
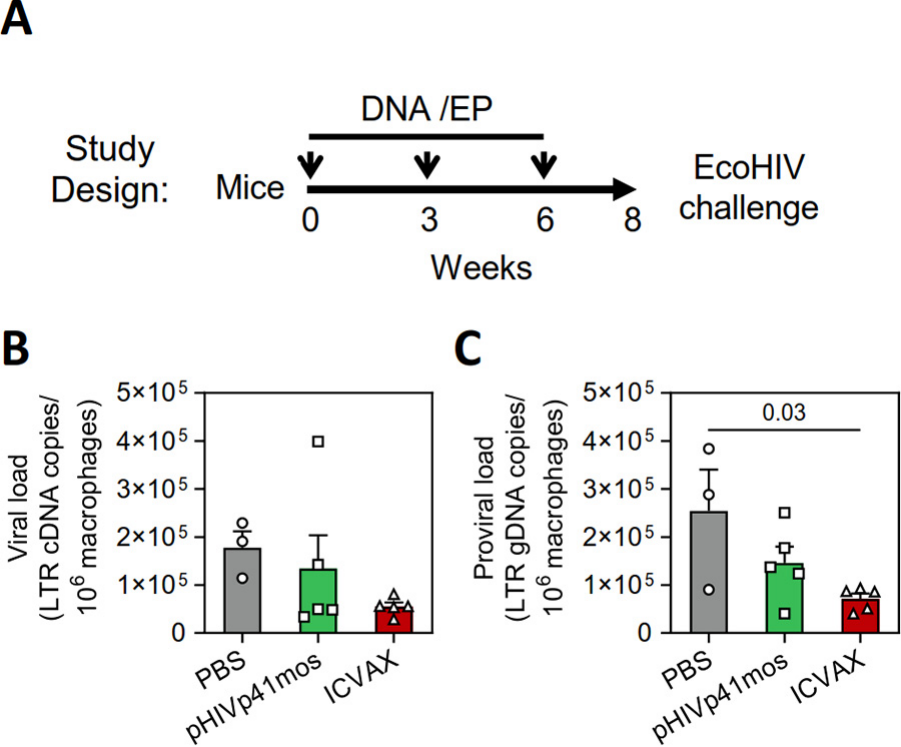
Efficacy of ICVAX vaccination against EcoHIV challenge in mice. (A) Schematic showing the immunization schedule for the DNA vaccines tested. In brief, BALB/c mice were immunized with 100 μg of the PD1-based ICVAX vaccine, the conventional pHIVp41mos vaccine, or PBS three times in 3-week intervals via i.m./EP. At 2 weeks after the last immunization, mice were challenged intraperitoneally with 5 × 10^5^ pg of p24 EcoHIV. Viral loads were then measured in peritoneal macrophages at 7 days post viral challenge. (B) EcoHIV cDNA and (C) genomic DNA (gDNA) viral loads in peritoneal macrophages from vaccinated mice 7 days after viral challenge.

A murine EcoHIV infection model that is relevant to HIV-1 infection was then used to investigate whether the adaptive immunity elicited by the ICVAX vaccine would induce antilentiviral protection. EcoHIV is a chimeric lentivirus that expresses the ecotropic murine leukemia virus envelope protein from an HIV-1 viral genome and replicates efficiently in mice (28). Here, groups of BALB/c mice (*n *= 5) vaccinated with ICVAX, pHIVp41mos, or PBS alone, were challenged intraperitoneally with 5 × 10^5^ pg p24 EcoHIV (29) 2 weeks after the third immunization (Fig. 7A). Since EcoHIV viral and proviral genome can be detected in spleen and peritoneal cells, with peritoneal cells having a higher viral loads per same number of cells and the presence of inducible replication-competent viruses in peritoneal macrophages (28, 30), the amount of EcoHIV was measured at 7 days post EcoHIV challenge from peritoneal macrophages. Mice vaccinated with ICVAX showed decreasing trend in viral RNA and proviral DNA compartments compared to mice that received PBS alone, although statistical differences were not reached (Fig. 7B and C). In comparison, there was a large variation in the viral RNA loads among animals in the pHIVp41mos group, with proviral DNA that did not significantly differ from control mice. These results indicated that ICVAX provided improved protection against EcoHIV in a mouse model of HIV-1 infection.

### Immunogenicity of ICVAX in nonhuman primates.

**FIG 8:**
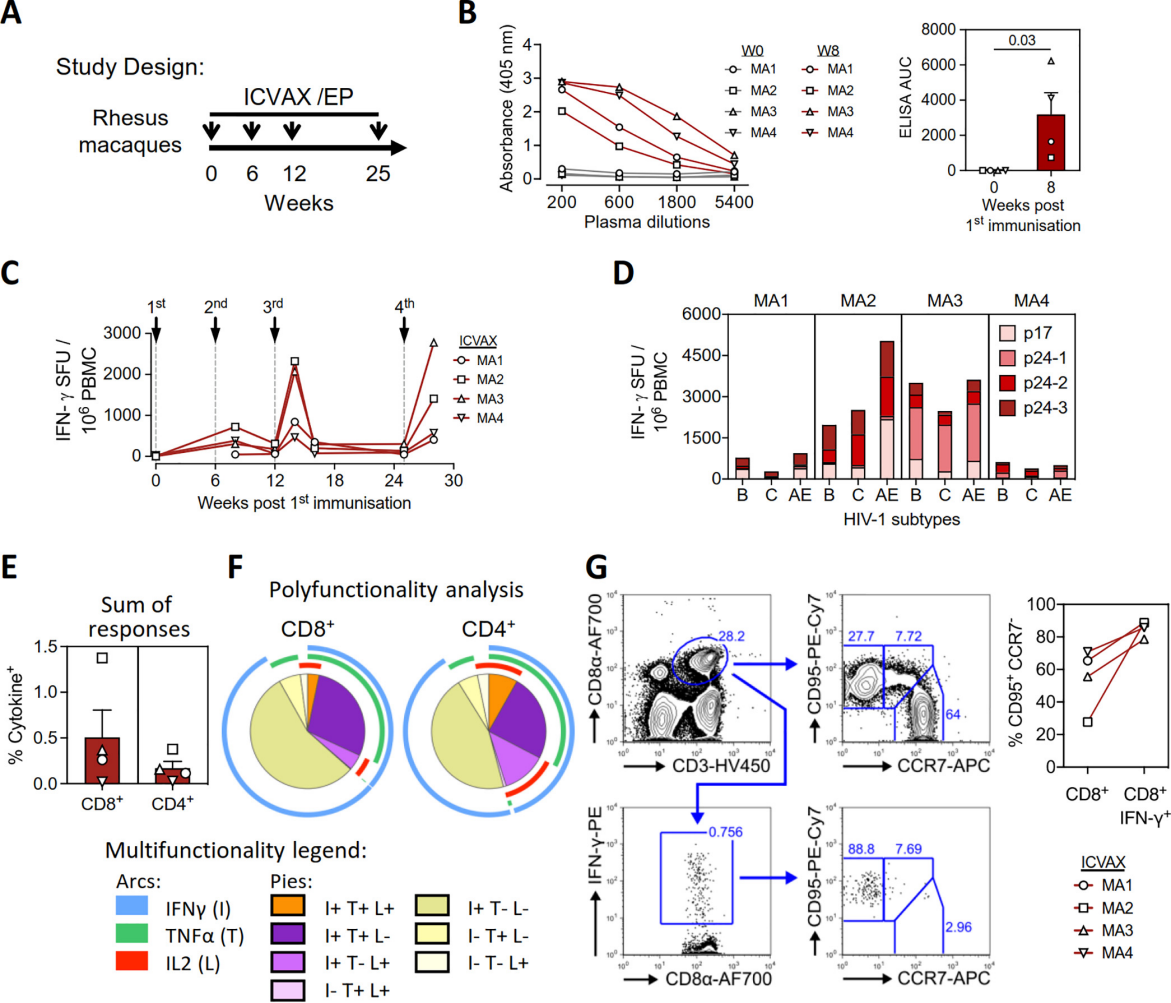
Immunogenicity of ICVAX vaccine in rhesus macaques. (A) Schematic showing the immunization schedule for ICVAX vaccination in 4 rhesus macaques. In brief, 4 macaques (MA1–MA4) were immunized with 2 mg ICVAX three times in 6-week intervals, followed by a boost vaccination 13 weeks after the third immunization via i.m./EP at 90V. Antibody and T cell responses were measured over time. (B) IgG antibody responses against HIV-1 Gag p24 were measured by ELISA. Anti-p24 antibodies in serial dilutions of sera were measured by absorbance (left panel) while anti-p24 titers were calculated as area under the curve (AUC; right panel). (C) Kinetics of T cell responses against HIV-1 subtype B Gag-p24 peptide pools, as measured in macaques by IFN-γ ELISpot assays. SFU, spot-forming units. (D) T cell responses against Gag-p41 peptide pools from HIV-1 subtypes B, C, or AE measured at 3 weeks after the 4th immunization, measured by IFN-γ ELISpot assays. (E) Frequencies of CD8^+^ and CD4^+^ T cells specific for HIV-1 subtype B Gag-p41, measured by intracellular cytokine staining at 3 weeks after the 4th immunization. (F) Polyfunctionality analysis of CD8^+^ (left panel) and CD4^+^ T cells (right panel) react against the HIV-1 subtype B Gag-p41 peptide pool. (G) Representative flow cytometry plots showing the gating strategy to identify CD95^+^CCR7^+^ and CD95^+^CCR7^–^ CD8^+^ T cells (left panels). The right panel compares the frequencies of effector CD95^+^CCR7^–^ cells in the whole CD8^+^ T cell population and the responding CD8^+^IFN-γ^+^ population, after *ex vivo* stimulation of PBMCs collected at 3 weeks after the 4th immunization with the HIV-1 subtype B Gag-p41 peptide pool.

In the following experiments, the ICVAX vaccine was tested in a nonhuman primate model that is more relevant to humans. Here, ICVAX immunogenicity was the main focus, and no other vaccine groups were included for comparison due to scarcity of rhesus monkeys. Four female rhesus macaques of Chinese origin (MA1-MA4) were immunized three times with 2 mg ICVAX via i.m./EP at 6-week intervals, followed by a fourth vaccination 13 weeks after the third immunization (Fig. 8A). Anti-p24 IgG antibody responses were measured from the sera of the macaques at an early time point, at 8 weeks post first vaccination, i.e., 2 weeks post second vaccination, to confirm the success of the vaccination (Fig. 8B).

The kinetics of Gag-specific T cell responses induced by ICVAX were measured over a 30-week period. Based on the IFN-γ ELISpot assays conducted with PBMC samples from the vaccinated macaques at various time points, we found that T cell responses against the HIV-1 subtype B Gag-p24 peptide pool were induced to higher levels upon the third vaccination (Fig. 8C). The responses reduced to lower yet detectable levels during the memory phase measured at 3 months after the third vaccination and, importantly, were again reboosted by the fourth vaccination by 5–10 fold (Fig. 8C). This result demonstrated the potential for repeated homologous immunization with ICVAX to restimulate antiviral T cell responses.

The breadth and cross-reactivity of the induced T cell responses were then evaluated at 3 weeks after the fourth immunization. PBMC were stimulated with 4 peptide pools covering the Gag-p41 antigens from HIV-1 subtypes CRF01_AE, followed by IFN-γ ELISpot assays (Fig. 8D). Although the magnitude of detected responses varied across macaques, all the peptide pools tested were immunogenic in each of the vaccinated macaques, illustrating that ICVAX elicited broad T cell responses that covered multiple regions on Gag-p41. Moreover, ICVAX-induced T cell responses were cross-reactive against the three HIV-1 subtypes tested, illustrating the capacity of induced T cells to recognize multiple viral variants.

ICS assays were also carried out to measure T cell polyfunctionality elicited by ICVAX in rhesus macaques. Responding T cells were readily identified at 3 weeks after the fourth immunization in both CD8^+^ and CD4^+^ T cell populations from the vaccinated macaques, after *ex vivo* stimulation with the HIV-1 subtype B Gag-p41 peptide pool (Fig. 8E). The majority of the responding T cells produced IFN-γ. The presences of IFN-γ^+^TNF-α^+^IL-2^+^, IFN-γ^+^TNF-α^+^, and IFN-γ^+^IL-2^+^ T cells was also detected in both CD8^+^ and CD4^+^ T cell populations (Fig. 8F). Overall, the polyfunctional subsets represented at least 21% and 38% of the responding CD8^+^ and CD4^+^ T cell populations, respectively (Fig. 8F).

Effector memory CD8^+^ T cells can migrate into peripheral tissues and possess better effector functions than central memory T cells (31). Effector memory CD8^+^ T cells are important in effective suppression of HIV-1 replication in elite controllers (32) and SIVmac251 replication in Indian-origin rhesus macaques receiving a CMV-vectored AIDS vaccine (33–35). We thus explored the memory differentiation of the responding T cell subsets induced by ICVAX in macaques. The CD95 and CCR7 markers were used to distinguish effector memory (CD95^+^CCR7^–^) and central memory subsets (CD95^+^CCR7^+^) (36). Interestingly, we found that the effector memory subset was enriched within the responding IFN-γ^+^CD8^+^ T cell population, when compared to the whole CD8^+^ T cell population (Fig. 8G).

**FIG 9:**
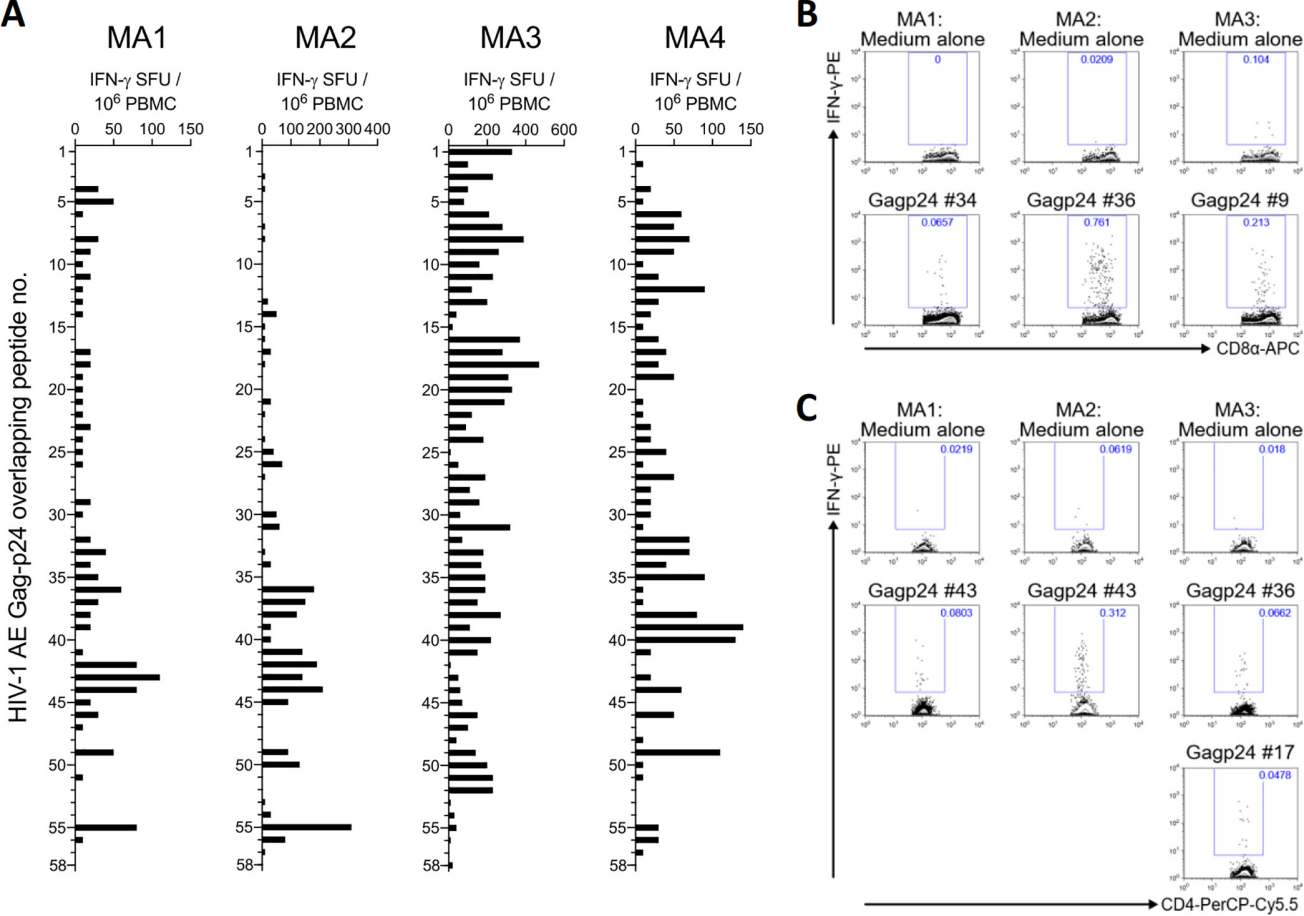
T cell epitope mapping of ICVAX-immunized rhesus macaques. (A) IFN-γ ELISpot responses against individual peptides spanning the Gag-p24 antigen of HIV-1 subtype AE. T cell responses induced in the ICVAX-vaccinated rhesus macaques at 2 weeks after the second immunization are reported. SFU, spot-forming units. (B–C) Flow cytometry plots demonstrating the reactivity of selected Gag-p41 peptides in the CD8^+^ T cell (B) and CD4^+^ T cell populations (C), as measured by intracellular cytokine staining using PBMC samples collected at 3 weeks after the 4th immunization.

We then mapped the specific T cell epitopes recognized in ICVAX recipient macaques to better evaluate the breadth of T cell responses, another parameter shown to be important for HIV-1 control (22, 37). IFN-γ ELISpot assays were first conducted to evaluate T cell reactivity of the four vaccinated macaques against individual overlapping 15mer peptides spanning the HIV-1 subtype AE Gag-p24 antigen (Fig. 9A). The results indicated that ICVAX-induced T cells did not preferentially target a particular region in the target antigen, but rather covered multiple regions that varied depending on individual animals. This likely reflects the diverse genetic backgrounds of the macaques tested, and also suggests that the mosaic antigen designed allowed effective antigen processing and presentation of multiple Gag regions (38). We further deconvoluted T cell responses to determine whether some of the identified epitopes were recognized by CD8^+^ or CD4^+^ T cells using ICS. Because of limited availability of specimen, only a few individual peptides were tested for each animal. CD8^+^ and CD4^+^ T cell epitopes were successfully identified for macaques MA1–MA3 (Fig. 9B and C), while no epitopes were determined from macaque MA4 in this experiment. Interestingly, the Gag-p24 peptide #43 was recognized by CD4^+^ T cells from MA1 and MA2 (Fig. 9C). This peptide consisted of the amino acid sequence VDRFYKTLRAEQATQ, a sequence that was homologous to the immunodominant CD4^+^ T cell epitope Gag-293 commonly recognized in elite controllers (39). This suggests that ICVAX may induce responses against protective T cell epitopes to exert protection.

### Evaluation of T cell responses induced by GMP-grade ICVAX in nonhuman primates.

**FIG 10:**
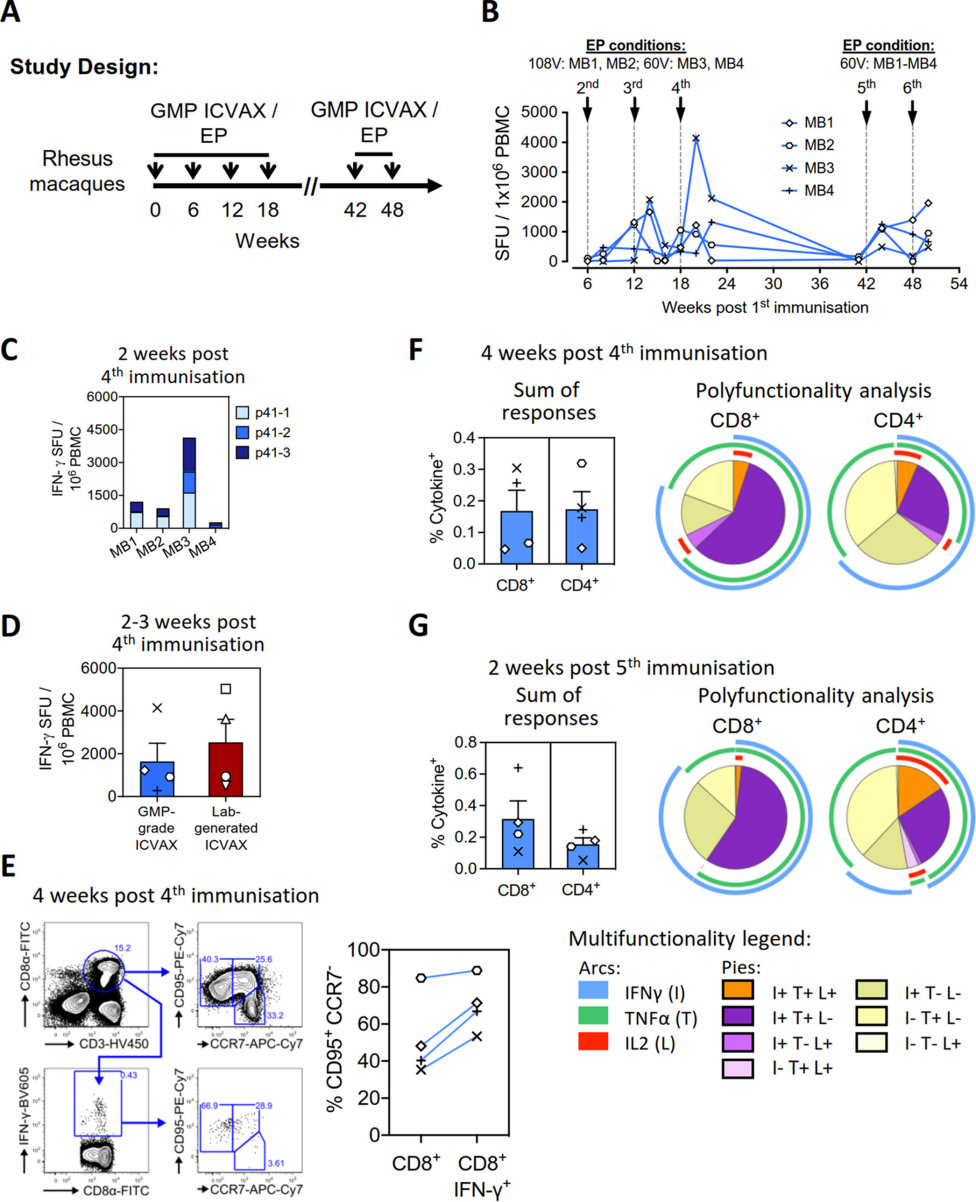
Immunogenicity of the GMP-g ICVAX vaccine in rhesus macaques. (A) Schematic showing the immunization schedule with the GMP-grade ICVAX in a new cohort of 4 rhesus macaques. In brief, 4 macaques (MB1–MB4) were immunized with 2 mg GMP-grade ICVAX four times at 6-week intervals, followed by two boost vaccinations at 42 and 48 weeks after the first immunization. The different voltages used for i.m./EP vaccination are reported on the figure. T cell responses were measured over time. (B) Kinetics of T cell responses in the macaques against the HIV-1 subtype AE Gag-p41 peptide pools, as measured by IFN-γ ELISpot assays. SFU, spot-forming units. (C) ELISpot-detected T cell responses against different peptide pools spanning HIV-1 subtype AE Gag-p41 from the vaccinated macaques at 2 weeks post 4th immunization. (D) The comparison of acute T cell responses against the HIV-1 subtype AE Gag-p41 induced by the GMP-grade and the laboratory-generated ICVAC at 2–3 weeks post 4th immunization, measured with IFN-γ ELISpot assays. (E) Representative flow cytometry plots showing the gating strategy to identify CD95^+^CCR7^+^ and CD95^+^CCR7^–^ CD8^+^ T cells (left panels). The right panel compares the frequencies of effector CD95^+^CCR7^–^ cells in the whole CD8^+^ T cell population and the responding CD8^+^IFN-γ^+^ population 4 weeks post 4th immunization. (F) Sum of T cell responses to the HIV-1 subtype AE Gag-p41 peptide pool at 4 weeks after the 4th immunization, as measured by intracellular cytokine staining (left panel). Polyfunctionality analysis of CD8^+^ (middle panel) and CD4^+^ T cell responses (right panel) were also analyzed. (G) Sum of T cell responses to the HIV-1 subtype AE Gag-p41 peptide pool at 2 weeks after the 5th immunization, as measured by intracellular cytokine staining (left panel). Polyfunctionality analysis of CD8^+^ (middle panel) and CD4^+^ T cell responses (right panel) were also analyzed.

We have recently demonstrated the efficacy of a rhesus PD1-based DNA vaccine in suppressing high dose simian–human immunodeficiency virus replication in Chinese-origin rhesus macaques (17). Because of these promising findings, a large-scale production of ICVAX vaccine was performed in a GMP-certified facility for future clinical trials. The immunogenicity of the GMP-grade product was subsequently evaluated in rhesus macaques. As ICVAX is designed as a therapeutic HIV-1 vaccine that may have to be repeatedly administered to infected patients, we examined the T cell immunogenicity of the vaccine in a biannual injection schedule to determine the antigen-specific T cell reactivity induced by homologous vaccination. A second cohort of four Chinese-origin rhesus macaques (MB1–MB4) were immunized with the GMP-grade ICVAX vaccine, first for 4 times at 6-week intervals, followed by boost vaccinations at 24 and 30 weeks after the initial round of vaccinations (Fig. 10A). Because a shortened interval between the 3rd and 4th vaccination induced better T cell responses in our rhesus PD1-based DNA vaccine study (17), the shorted initial vaccination regimen was followed in this macaque cohort. We also aimed at optimizing the electroporation protocol to improve the delivery of the DNA vaccine. Two different electroporation voltages were tested in the first four vaccinations, each in two macaques: 108V in macaques MB1 and MB2, and 60V in macaques MB3 and MB4. We used 108V in our previous rhesus PD1-based DNA vaccine efficacy study (17), while 60V was used in our published murine PD1-based DNA vaccine studies (15, 16).

Similar to the macaque study with ICVAX produced in our own laboratory, the GMP-grade ICVAX was highly immunogenic, as measured by T cell responses induced against the HIV-1 subtype AE Gag-p41 antigen (Fig. 10B). The series homologous ICVAX vaccinations progressively enhanced the Gag-specific T cell responses during the initial treatment period. Because a significant variation in responses was detected in the four animals, we could not draw conclusions on the effect of the two electroporation voltages applied regarding the T cell immunogenicity. The laboratory-generated ICVAX and GMP-grade vaccine showed a comparable immunogenicity to induce acute T cell responses across various Gag-p41 regions (Fig. 10C and D). Importantly, the homologous ICVAX boost vaccinations administered at 24 weeks after the completion of initial vaccination schedule successfully reactivated the Gag-p41-specific effector memory T cell responses (Fig. 10B and E), demonstrating the feasibility of ICVAX vaccination in a biannual treatment regimen.

The phenotypes of the antigen-specific CD8^+^ T cells measured at 4 weeks post fourth vaccination indicated that effector-memory CD8^+^ T cell responses were induced by the GMP-grade ICVAX (Fig. 10E), similar to responses elicited by the laboratory-generated ICVAX vaccine (Fig. 8G). The T cell polyfunctionality was also measured at 4 weeks after the fourth immunization (week 22) and 2 weeks after the fifth immunization by ICS (Fig. 10F and G). The overall CD8^+^ and CD4^+^ T cell responses against HIV-1 subtype AE Gag-p41 antigen were comparable between both time points, confirming effective T cell reactivation after a delayed boost vaccination. The frequencies of polyfunctional T cells that expressed IFN-γ/TNF-α/IL-2, IFN-γ/TNF-α, or IFN-γ/IL-2 were similar at the two time points tested in both the responding CD8^+^ and CD4^+^ T cell populations after *ex vivo* subtype AE Gag-p41 peptide pool stimulation. Our results demonstrated that the magnitude and polyfunctionality of antigen-specific effector-memory T cells induced by the GMP-grade ICVAX vaccine were well maintained even after a delayed boost vaccination. Thus, administering ICVAX every 6 months resulted in a highly immunogenic vaccination regimen.

## DISCUSSION

Sterilizing HIV-1 cure has been achieved in only a very limited number of patients by allogeneic transplantation of haemopoietic stem cells that did not express CCR5, the coreceptor for HIV-1 entry (40–42). The treatment procedure involved is very complex, and the mechanisms underlying complete HIV-1 eradication from these rare individuals are still under investigation (43). A functional HIV-1 cure, in which HIV-1 viremia is suppressed in the long term by the immune system, represents an alternative approach to induce viral control in the absence of lifelong antiretroviral therapy (44). In this study, we demonstrated the immunogenicity and the potential efficacy of the PD1-based ICVAX DNA vaccine in mice. The use of a soluble PD1 domain construct significantly increased the T cell immunogenicity and cross-reactivity of the mosaic DNA vaccine as shown in the mouse model. Moreover, ICVAX vaccination led to suppression of EcoHIV proviral load in mice. In the rhesus macaque model, ICVAX also elicited cross-reactive effector memory T cell responses, which have been associated with better HIV-1 control by previous studies (17, 45, 46). Importantly, the T cell responses induced by the initial series of vaccinations were successfully reactivated upon homologous boost vaccinations 6 months later. These results support further evaluation of ICVAX as a therapeutic vaccine to induce a functional HIV-1 cure.

The better immunogenicity elicited in mice by ICVAX demonstrated that the PD1 targeting strategy was more superior than pHIVp41mos lacking the soluble PD1 domain. The data supported further evaluation of ICVAX alone in nonhuman primates (NHP) rather than including the pHIVp41mos to reduce experimental cost. To better recapitulate the efficacy of ICVAX for potential clinical application, we further evaluated the vaccine-induced HIV Gag-p41 specific immunogenicity with the GMP-grade version of ICVAX in NHP. Two separated NHP studies were conducted in the current study, one focused on lab-prepared ICVAX while the other focused on the ICVAX prepared in a GMP facility. Both grades of the vaccine induced similar cross-reactive polyfunctional T cell immune responses against the encoded HIV-1 p41 antigen, indicating the immunogenicity of the vaccine and its readiness for clinical trial use. The second NHP study also demonstrated the T cell immunogenicity of the GMP-grade ICVAX vaccine as a homologous booster vaccine. In fact, T cell response induced by ICVAX vaccine in the current NHP studies had a highly similar phenotype as that induced by the rhesus PD1-based vaccine, which provided sustained SHIV control in macaques in our recently published study (17). Overall, these data in NHP together illustrate the potential of ICVAX for clinical use. It should also be noted that because all Chinese-origin rhesus macaques used in the current studies were out-bred, their genetic backgrounds would be diverse, similar to the human population. It was expected that the immune responses induced by the ICVAX vaccine in NHP would vary among the tested animals. To fully understand the potency and efficacy of the ICVAX vaccine, conducting human clinical trials to examine whether the vaccine could induce cross-reactive polyfunctional T cells that can actively suppress viral replication is needed.

Various strategies aiming to achieve a functional HIV-1 cure are being developed. One of these involves the use of broadly neutralizing antibodies (bNAb) against HIV-1 to suppress viral replication after cART interruption (47–49). Similarly, anti-CD4 antibodies, including UB-421, which blocks the HIV-1 binding site on the CD4 molecule (50), could also be used to suppress viral replication without cART. It should be noted that antibody-resistant viral variants are likely to emerge during bNAb monotherapy (47, 48), and that combination therapy with multiple antibodies might be required (49). In addition, repeated injections of these biologics would be required to maintain an effective antibody concentration in patients. Another approach using recombinant adeno-associated viral vector to facilitate long-term gene transfer of multiple bNAb constructs has shown some promising results in viral suppression in HIV-1-infected humanized mice and SHIV-infected rhesus macaques (51, 52). Before further clinical evaluation, there are still hurdles in terms of vector and transgene immunogenicity and safety that need to be addressed. In comparison, therapeutic vaccination with DNA constructs such as ICVAX, has the potential to induce antiviral immune responses that actively suppress HIV-1 replication, and represents an attractive approach that would require fewer injections. In addition, ICVAX could also be used in a combined immunotherapy strategy.

Several therapeutic HIV-1 vaccines have been evaluated in clinical trials. An early trial examined the efficacy of a replication-defective adenovirus type 5 vector expressing HIV-1 Gag antigen in patients living with HIV-1 (53). Although the vaccination only slightly enhanced the CD4^+^ T cell response against Gag and did not delay viral rebound upon cART interruption, an association between the antigen-specific response and lower viremia level after treatment interruption was detected (53). Autologous DC loaded with lipopeptides carrying HIV-1 antigens have also been tested as a therapeutic vaccine in patients, with an increase in frequencies of polyfunctional CD4^+^ and CD8^+^ T cells and the breadth of T cell responses (54). More recently, various novel antigen constructs expressed from heterologous viral vectors involving chimpanzee, or less common adenovirus serotypes or poxvirus vectors, have been or are being tested in clinical trials (8, 9, 12, 55). The immunogens evaluated were designed based on T cell epitopes recognized by elite and viraemic controllers (12, 56), highly conserved regions on HIV-1 antigens (9, 55), or mosaic antigen (8). The first two antigens, namely, HTI (12) and HIVconsv (9), aimed to generate T cell responses that would be focused on epitopes associated with HIV-1 protection. In particular, the mosaic antigenic constructs are designed to cover a maximum number of PTE from diverse viral sequences (18). The vaccination regimen using adenoviral/poxviral vectored vaccines encoding trivalent mosaic HIV-1 antigens resulted in the induction of potent anti-HIV-1 immune responses, with a slight delay of viral rebound upon cART interruption (8). Overall, these promising results demonstrate the potential of T cell therapeutic vaccines to control HIV-1 replication, but further development seems necessary to improve the clinical benefits of the vaccines.

The induction of broad antiviral T cell immunity needed to eliminate HIV-1 infected cells would be an important attribute of an effective T cell vaccine (57). Due to the combination of a soluble human PD1 domain and a mosaic antigen design, our ICVAX vaccine successfully induced high frequencies of polyfunctional T cells in a mouse model, confirming our previous studies using murine PD1-based vaccines (15, 16). In fact, our previous publication showed that PD1-fused antigen allowed better binding to DC and induced better DC activation, compared to non-PD1 fused antigens (15). Although a range of cell types express PD1 ligand, PD-L1 is constitutively expressed on immature DCs. The soluble PD1 can therefore effectively deliver the mosaic antigen to DC for efficient antigen presentation during T cell priming (58). In this study, ICVAX enhanced T cell responses against the Gag-p41 antigen from HIV-1 subtypes B and AE in mice, while the response against the subtype C’s Gag-p41 antigen induced by ICVAX was similar to that of the nontargeting pHIVp41mos vaccine 2 weeks after completion of the whole vaccination course. Our previous publication also showed a very good T cell response against Gag p24 in mice using the PD1-targeting strategy at 2 weeks after the last vaccination (15). T cell response against the Gag-p41 of subtype C might have been peaked when pHIVp41mos was used, and so further improvement of antigen targeting with the inclusion of the PD1 domain could not further enhance the adaptive immune response. Further investigation is needed to understand this observation. Furthermore, ICVAX induced cross-reactive, polyfunctional effector memory T cell responses that recognized multiple regions on the targeted Gag-p41 antigen in rhesus macaques. These properties recapitulate those of effector-memory T cell responses induced by the rhesus PD1-based DNA vaccine, which has shown efficacy at inducing long-term SHIV suppression in rhesus macaques following viral challenge in our recent study (17). The current *in vivo* findings therefore provide evidence support to further evaluate polyfunctional cross-reactive T cell responses elicited by ICVAX, and its efficacy in humans, by proceeding into clinical trial. Taken together, these findings warrant the development of ICVAX as a therapeutic HIV-1 vaccine.

It has been suggested that matching vaccine antigens to the virus present in the patients could be necessary for antiviral control (8). This key parameter has been evaluated in our patient cohorts. T cell cross-reactivity toward the mos1 and mos2 antigens encoded by the ICVAX vaccine was detected in nearly all patients tested. This suggests that the mosaic antigens encoded by ICVAX are likely to be recognized by patients’ T cells, and may have the potential to reactivate antiviral immune responses in patients. Interestingly, patients receiving a combination treatment of 2 NRTI + 1 INSTI had a higher IFN-γ response against mos2 than patients receiving 2 NRTI + 1 NNRTI, and this might possibly be due to the suppression of CD8^+^ T cell activation with prolonged NNRTI treatment (59, 60). Furthermore, T cell responses against Gag293, a highly immunodominant CD4 epitope associated with elite controller (61), were elicited by ICVAX in rhesus macaques. Higher CD4^+^ T cell functionality against Gag293 has previously been identified in HIV-1 controllers (37), suggesting a potential role for such T cell response in promoting viral suppression. Although the protective role of this T cell epitope in rhesus macaques is less characterized, this interesting finding of ICVAX-induced Gag293-specific T cell response, together with enhanced breadth to multiple epitopes, further supports the immunotherapeutic potential of the vaccine.

Antivector immunity is another issue that not only curbs the immunogenicity of the expressed specific antigen, but might also enhance the risk of infection (62, 63). As a DNA vaccine, ICVAX does not pose major issues related to preexisting antivector immunity. Importantly, the rhesus PD1-based DNA vaccine boosted effector-memory T cell responses without activation of latent SHIV after viral suppression. Taking another step forward, we demonstrated here that ICVAX could also be administered repeatedly in a biannual setting to amplify the anti-Gag-p41 effector-memory T cell responses. This finding suggests that homologous boost vaccination with ICVAX could be performed repeatedly if necessary for HIV-1 patients’ functional cure. Owing to the differences in Gag-p41 sequences between HIV-1 and SIV or between human and macaque, it was not possible to carry out SIV/SHIV or HIV-1 challenge for vaccinated macaques in this study. In fact, our previous study already demonstrated that the same PD1-based approach could effectively suppress SHIV when delivering SIV Gag-p27 antigen to dendritic cells for priming specific board T cell and antibody responses (17). However, several features of EcoHIV-infected mice are different from HIV-1-infected patients, and the efficacy of ICVAX in the EcoHIV model should be carefully interpreted. For example, infected cART-untreated mice remained immunocompetent with stable CD4^+^CD8^+^ ratio at 1 and 4 months after infection, resembling features of patients undergoing effective cART (30). Persistent viral expression was also found in infected macrophages of EcoHIV-infected mice (30). These differences would affect the kinetics of *in vivo* viral control, and the efficacy of ICVAX should therefore be further evaluated when proceeding into clinical trial. Nevertheless, data from the EcoHIV-1 model provided an essential proof of concept that the newly designed mosaic antigens linked to the PD1 domain could elicit a better control, especially in terms of polyfunctional antigen-specific T cell responses against HIV-1 *in vivo*. This important evidence-based support allowed us to further evaluate the GMP-graded vaccine in nonhuman primates, and the potential of developing the vaccine for proceeding into future clinical trial.

Lastly, immunotherapeutic vaccination alone might not completely eliminate viral reservoirs, which determines the speed of viral rebound after cART interruption (64). Combination therapies with repeatable vaccinations and LRA could allow a more effective destruction of latently infected cells that would be reactivated in a controlled manner (65). Although early clinical trials of the “shock and kill” strategy showed mixed results (14, 55, 66), the recent development of more potent LRA and vaccine constructs may allow further improvement of immunotherapeutic regimens (13, 67). ICVAX, therefore, should be evaluated in combination with other promising strategies for HIV-1 functional cure.

## MATERIALS AND METHODS

### Animals and patients.

All mouse experiments were conducted at the University of Hong Kong and were approved by its Committee on the Use of Live Animals in Teaching and Research. Six- to eight-week-old female BALB/c mice were immunized with 100 μg of ICVAX, pHIVp41mos, or PBS for three times at 3-week intervals (*n *= 3–4/group) intramuscularly (i.m.) with electroporation (EP) at 60V with the second-generation TERESA-EPT Gene Delivery Device (Shanghai Teresa Healthcare). For the nonhuman primate studies, two cohorts of Chinese-origin rhesus macaques were tested. The macaque studies were approved by the Institutional Animal Care and Use Committee of the Department of Veterinary Medicine at Foshan University. Welfare and condition of all animals were closely monitored according to approved protocol. In the first macaque cohort, four female macaques were immunized with 2 mg laboratory-generated ICVAX via i.m./EP three times at 6-week intervals, followed by a boost vaccination at 13 weeks post third immunization, with EP voltage set at 90V (Fig. 8A). In the second cohort, four female macaques were immunized with 2 mg GMP-grade ICVAX via i.m./EP four times at 6-week intervals with EP at either 60V or 108V, followed by ICVAX revaccination at 24 and 30 weeks post first immunization with EP at 60V (Fig. 10A). PBMC and plasma were isolated at various time points for immunological testing. Regarding patient study, all patients gave informed consent, and procedures were reviewed and approved by the Ethics Review Committee of Shenzhen Third People’s Hospital and the Joint Chinese University of Hong Kong-New Territories East Cluster Clinical Research Ethics Committee. A total of 83 HIV-1 infected patients, including 44 cART-naive and 39 cART-treated patients, were tested in this study. Peripheral blood mononuclear cells (PBMC) and sera were collected from these patients. Clinical data and demographic of patients are shown in Tables 1 and 2.

### Vaccine constructs.

The HIV-1 gp41 mosaic antigens mos1 and mos2 were designed *in silico* based on >500 HIV-1 viral strains circulating in China, covering HIV-1 subtypes B, CRF01_AE, and CRF07/08_BC (19, 23). A codon-optimized DNA sequence encoding the mosaic HIV-1 mos1 and mos2 antigens, with a flexible (G_4_S)_3_ linker in between, was synthesized by GenScript. This DNA fragment was used as a template for the amplification of the bivalent mosaic antigen sequence by PCR using PrimeSTAR HS DNA polymerase (TaKaRa) with the following primers: 5′-CCGGAATTCCGGGGAGGCGGGGGAAGTGGAGGAGGAGGATCCGGAGGAGGAGGAAGCATGGGGGCAAGAGCCTCC-3′ and 5′-CCGGCGCGCCGTTTAAACAAAGCTCCGCTCGAGCGGTTATCACAGCACTCTGGCTTTATG-3′. The PCR product was purified and digested with the EcoRI and PmeI restriction enzymes (Fermentas), and cloned into an EcoRI/PmeI linearized pVAX plasmid encoding a codon-optimized human soluble PD1 domain with a tissue plasminogen activator (tPA) signal peptide at the N terminus using T4 DNA ligase (TaKaRa) to generated the ICVAX vaccine. In parallel, another aliquot of the mosaic PCR product was digested with EcoRI and XhoI enzymes (Fermentas), and was cloned into an EcoRI/XhoI linearized pVAX plasmid encoding only a tPA signal peptide using T4 DNA ligase to generate the pHIVp41mos vaccine. The DNA vaccine sequences were confirmed by Sanger sequencing. The plasmid pmsPD1-HIVp24fc, a DNA vaccine that encodes a murine soluble PD1-fused HIV-1 p24 antigen, was described previously (15). The laboratory-grade DNA vaccines were generated using EndoFree Plasmid Giga Kit (Qiagen). The GMP-grade ICVAX vaccine was manufactured by the Guangzhou BaiDi Biological Pharmaceutical Company, Limited. Expression of the immunogens were confirmed by Western blotting using the anti-HIV-1 Gag-p24 antibody clone 13-H12-5C (NIH AIDS Reagents Program). For detection of ICVAX and pHIVp41mos in the culture supernatant of transfected 293T cells, anti-HIV-1 Gag-p17 antibody clone 17-1 (Abcam) was used.

### *In vitro* binding of immunogens to PD1 ligands.

293T cells were transfected with the ICVAX, pHIVp41mos, or pmsPD1-HIVp24fc plasmids using polyethylenimine. Two to 3 days later, supernatants were collected and were used as the sources of recombinant immunogens for testing their binding ability to PD1 ligands expressed on transiently-transfected 293T cells, as described previously (15).

### EcoHIV challenge.

EcoHIV, a chimeric HIV carrying an ecotropic murine leukemia virus envelope, efficiently replicates in mice (28). Vaccinated mice were challenged intraperitoneally with 5 × 10^5^ pg EcoHIV 2 weeks after the third immunization. Seven days after the EcoHIV challenge, peritoneal macrophages were isolated from mice. RNA and DNA viral loads were determined by qRT-PCR as we described previously (19).

### Antibody response assays.

Gag-specific antibody responses in sera or plasma were assessed by ELISA against the HIV-1 Gag-p24 antigen (Abcam) as previously described (19).

### T cell response assays.

T cell immune responses were examined by interferon-γ (IFN-γ) ELISpot, intracellular cytokine staining (ICS), or tetramer staining assays. In ELISpot assays, 1–2 × 10^5^ PBMC from HIV-1 patients, splenocytes from mice, or PBMC from rhesus macaques were stimulated with 1 μg/mL of 15-mer overlapping peptide pools spanning the mos1 or mos2 antigens (GL Biochem [Shanghai] Limited), and Gag-p24 or Gag-p41 antigens from HIV-1 subtype AE, B, or C (NIH AIDS Reagents Program), and the T cell responses were determined using the relevant IFN-γ ELISpot kits (U-Cytech or MabTECH) (17, 19). In ICS assays, 0.5–2 × 10^6^ human PBMC, mouse splenocytes, or macaque PBMC were stimulated with 1 μg/mL of 15-mer overlapping mos1 peptide pool or Gag-p41 peptide pools from HIV-1 subtypes B, C, or AE in the presence of 0.5 μg/mL of anti-CD28 and anti-CD49d antibodies (Biolegend). After 2-h incubation at 37°C with 5% CO_2_, Brefeldin A (Sigma-Aldrich) was added, and the cells were further incubated overnight. Cells were then surface stained, fixed, and permeabilized using the Cytofix/Cytoperm kit (BD Biosciences), and stained for intracellular cytokines. The following antibodies were used to detect responses in human, mouse, or macaque samples: anti-CD3, anti-CD4, anti-CD8α, anti-CD95, anti-CCR7, anti-IFN-γ, anti-tumor necrosis factor α (TNF-α), and anti-interleukin 2 (IL-2) (BD Biosciences or Biolegend). Samples were acquired with the FACSAria III instrument (BD), and data were subsequently analyzed using FlowJo v9 or v10 software (BD). T cell polyfunctionality analysis was performed with SPICE 6 (68). For epitope mapping, responses toward individual 15-mers peptides (1 μg/mL) were determined using macaque PBMC with IFN-γ ELISpot assays, followed by ICS assays with selected antigenic peptides. In tetramer staining, H-2K^d^/AI tetramer (containing the Gag AMQMLKDTI peptide; Beckman Coulter) was used to determine the magnitude of CD8^+^ T cells specific for the immunodominant AI epitope from the HIV-1 Gag-p41 antigen in vaccinated BALB/c mice.

### Statistical analysis.

Statistical analyses of data were performed using Prism v7 (GraphPad). In mouse studies, significances of differences were determined by unpaired Student's *t* test between two treatment groups, or by ANOVA, followed by Tukey multiple-comparison test for comparisons between more than two groups. In macaque and human studies, after a D'Agostino-Pearson omnibus normality test, two-tailed Wilcoxon rank sum test, paired Wilcoxon test, or Kruskal-Wallis test followed by the Dunn’s multiple-comparison test were performed for the comparisons between two or more than two treatment groups. To compare clinical parameters between cART-naive or treated patients, Fisher’s exact test was used for discrete or categorical data while unpaired Student's *t* test was used for continuous data. For correlation of patient clinical data, the D'Agostino-Pearson omnibus normality test was used to determine normality, followed correlation analysis by Pearson correlation for normally distributed data and Spearman correlation for non-normally distributed data. Probability value (*p*) of smaller than 0.05 was considered as statistically significant.
